# Effects of Caffeine on Performances of Simulated Match, Wingate Anaerobic Test, and Cognitive Function Test of Elite Taekwondo Athletes in Hong Kong

**DOI:** 10.3390/nu14163398

**Published:** 2022-08-18

**Authors:** Fenghua Sun, Agatha Yi-Sum Siu, Kangle Wang, Borui Zhang, Man-Him Chan, Ka-Hon Chan, Pui-Sze Kong, Kei-Yee Man, Gary Chi-Ching Chow

**Affiliations:** Department of Health and Physical Education, The Education University of Hong Kong, Hong Kong SAR, China

**Keywords:** reaction time, fNIRS, anaerobic capacity, exercise performance, taekwondo, caffeine

## Abstract

This study aimed to investigate the effects of caffeine on performances of simulated match, Wingate Anaerobic Test (WAnT), and cognitive function test of elite taekwondo athletes. Ten elite taekwondo athletes in Hong Kong volunteered to participate in two main trials in a randomized double-blinded crossover design. In each main trial, 1 h after consuming a drink with caffeine (CAF) or a placebo drink without caffeine (PLA), the participants completed two simulated taekwondo match sessions followed by the WAnT. The participants were instructed to complete three cognitive function tests, namely the Eriksen Flanker Test (EFT), Stroop Test, and Rapid Visual Information Processing Test, at baseline, before exercise, and immediately after the simulated matches. They were also required to wear functional near-infrared spectroscopy equipment during these tests. Before exercise, the reaction time in the EFT was shorter in the CAF trial than in the PLA trial (PLA: 494.9 ± 49.2 ms vs. CAF: 467.9 ± 38.0 ms, *p* = 0.035). In the WAnT, caffeine intake increased the peak power and mean power per unit of body weight (by approximately 13% and 6%, respectively, *p* = 0.018 & 0.042). The performance in the simulated matches was not affected by caffeine intake (*p* = 0.168). In conclusion, caffeine intake enhances anaerobic power and may improve certain cognitive functions of elite taekwondo athletes in Hong Kong. However, this may not be enough to improve the simulated match performance.

## 1. Introduction

Taekwondo is a high-intensity combat sport that involves poomsae motions and sparring [1]. The total energy cost of taekwondo is predominantly borne by the aerobic system due to the high intensity but low frequency of pauses, step movements, and kick movements involved [2]. Therefore, a moderate to high level of cardio-respiratory fitness is required by taekwondo athletes [3]. However, how well a taekwondo athlete performs is primarily dependent on anaerobic power due to the importance of explosive movements on the lower limbs [4]. Performance in a taekwondo match may also be affected by cognitive factors, such as reaction time, motor time, information processing speed, and working and executive semantic memory [1]. In taekwondo, the competitive environment is dynamic, complex, and demands sustained high-quality cognitive processing. Thus, better cognitive performance (i.e., quicker processing time and more effective decision-making results) has the potential to improve taekwondo performance. Furthermore, only when an athlete achieved sustaining power output cooperating with efficient mental thoughts, tactical moves can be delivered in combat competition. Therefore, both anaerobic power and cognitive function are the most important factors influencing taekwondo performance.

The use of certain nutritional ergogenic aids has been suggested to improve specific combat skills and physical aspects such as anaerobic metabolism, while it seems that only caffeine has shown evidence of enhancing the performance of combat sports [5]. Caffeine is a common supplemental-nutritional substance that is frequently used to increase alertness and reduce fatigue among humans [6]. Recent data in Spain [7] suggested a moderate usage of caffeine in most sport specialties and more uses of caffeine in competition among athletes of individual sports or aerobic-nature sports. Although it is still unclear how many combat sports athletes consume caffeine before exercise, the effects of caffeine consumption on combat sports performance have been investigated a lot, possibly due to its potential ergogenic effects [5,8,9].

Two studies performed among elite jiu-jitsu athletes [10,11] suggested the ergogenic effects of a dose of 3 mg/kg body mass of caffeine on certain physiological and psychological indicators such as vertical jumps, power, fatigue perception, and endurance perception. The ergogenic effects of caffeine also include enhanced endurance capacity [12], anaerobic capacity [13], and neuromuscular function [14], and a recent umbrella review [15] has provided more consolidated evidence. However, it seems that the magnitude is generally greater for aerobic than anaerobic exercise. The ergogenic effects may be caused by the increased glycolytic dependence [8] and the stimulated central nervous system (CNS) [16], including the enhanced cognitive processes [17] and other cognitive tasks performance such as concentration levels, attentional focus, information recall, memory, and simple motor speed [18,19]. In suitable doses (e.g., 200 mg), caffeine can improve cognition attributed to the antagonizing ability of adenosine A1- and A2a-receptors in the CNS [20], as well as exert adenosine-mediated effects on dopamine-rich brain areas, which are responsible for executive function and alertness [21]. Another study found that in optimal doses, caffeine can enhance physiological arousal by increasing heart rate and blood pressure, therefore improving the executive control of visual attention [22].

The enhanced CNS functions after caffeine consumption may influence both cognitive and physical performance [23,24]. In combat sports, caffeine was found to benefit neuromuscular efficiency and improve reaction speed [25]. Furthermore, previous studies also suggested that caffeine supplements could improve mood [26] and reduce the feeling of fatigue [27], and all of these may also contribute to improved performance. Despite all these obvious benefits of caffeine consumption, in one previous systematic review [8], only six of nine studies have reported improved performance after caffeine consumption in combat sports. In one more recent meta-analytical study [9], caffeine consumption was found to increase the number of throws, offensive actions, and duration of offensive actions during combat sports. However, to the best of our knowledge, limited studies have investigated the potential ergogenic benefits of caffeine among taekwondo athletes. By using the bandal tchagui (five kicks) reaction time test, Santos et al. [24] found that caffeine ingestion reduced the reaction time in taekwondo events under a non-fatigued condition. In another study [28], caffeine ingestion increased glycolytic contribution but had no effects on performance or other subjective indicators. This may be caused by its antagonistic action on peripheral adenosine receptors, as well as the promoted catecholamine release to facilitate muscle glycogenolysis. Furthermore, the ergogenic effects of caffeine use may be influenced by many different factors including caffeine effects, daily habits, physiological factors, and even genetic factors [29]. Therefore, additional studies must be conducted to clarify the effects of caffeine ingestion on physical and cognitive performance in taekwondo athletes. As a newly developed technology, recently functional near-infrared spectroscopy (fNIRS) has been used to accurately measure the cortical hemodynamic response under different exercise conditions, which may be helpful for exploring the potential mechanism behind changes in cognitive function related to exercise [30]. The fNIRS is a sensitive non-invasive means of measuring changing levels of oxyhemoglobin (O_2_Hb) and deoxyhemoglobin (HHb) in the superficial layers of the cortex [31]. This measurement is important as cerebral perfusion to the prefrontal cortex may be an important mechanism linking exercise to improved executive function [32]. Although one recent study [33] has used fNIRS to investigate the effects of different coffee conditions on modulating executive function among healthy participants, to the best of our knowledge, fNIRS has yet to be used to investigate the effect of caffeine consumption on the cognitive function of elite taekwondo athletes. 

Therefore, the purpose of the present study was to investigate the effects of caffeine ingestion on the cognitive function of elite taekwondo athletes in Hong Kong and on their performance in a simulated taekwondo competition. The changes in the concentrations of the O_2_Hb and HHb in the brain prefrontal area during cognitive function tests were monitored through fNIRS. It was hypothesized that caffeine ingestion would not only enhance the simulated match performance but also improve performance in cognitive function tests.

## 2. Materials and Methods

### 2.1. Participants

A total of 14 elite taekwondo athletes from Hong Kong’s national taekwondo team were recruited for the present study. All the athletes had at least 5 years of Taekwondo training experience (6 ± 1 years). However, due to the COVID-19 pandemic and government coronavirus lockdowns and restrictions, only 10 participants (6 males, age: 27 ± 2 years, height: 174 ± 7 cm, and weight: 71 ± 11 kg; 4 females, age: 25 ± 3 years, height: 162 ± 6 cm, and weight: 58 ± 1 kg) completed all the experimental trials. All the participants signed a consent form and completed a health screen questionnaire before the experiment to ensure that they had no health and physical condition that would affect their ability to complete the study. The study procedures were approved by the Human Research Ethics Committee of The Education University of Hong Kong.

### 2.2. Study Design

**Figure 1:**
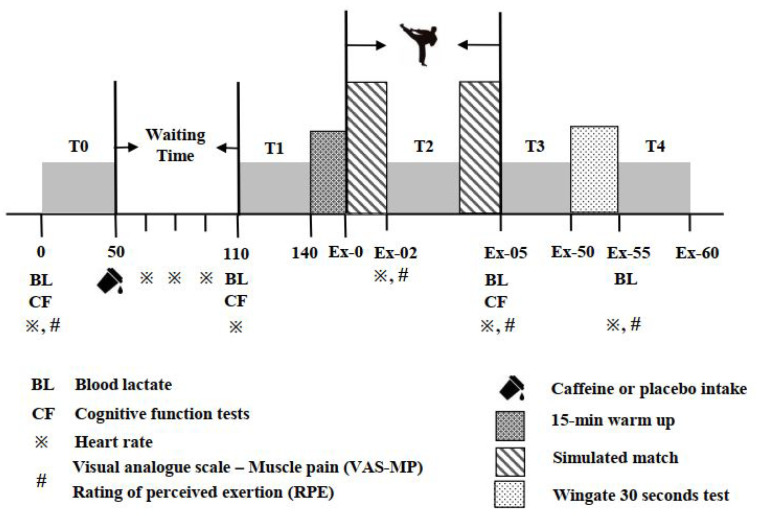
Illustration of experimental protocol.

All the participants participated in two experimental sessions in a randomized double-blinded crossover study design. The present study was conducted from 10 December 2019 to 5 July 2020, and the participants were paired according to their performance in their daily training sessions. Each pair of participants was required to attend the two experimental sessions together at the same time period, and each session lasted for approximately 3 h. The washout period between two sessions for each pair of participants was around 1 week. There were 4 training sessions per week during the experimental period. The total training hours were around 11 h/week including cardiorespiratory training (1 h), fitness training (1 h), strength training (2 h), skill performance (5 h), and simulated match (2 h). The menstrual cycle of female participants was not strictly controlled. However, they were required to not complete the experiments during their menses phase. In each pair, one participant was assigned to a placebo (PLA) trial and the other was to a caffeine (CAF) trial randomly in the first session, and they changed their trials in the second session accordingly. The experimental protocol for each session is displayed in Figure 1. The procedure adopted in both sessions was identical except for the liquid intake (either PLA or CAF) at the beginning of the test.

In the CAF trial, the participants ingested a drink with 200 mg of caffeine, whereas they ingested a placebo drink without caffeine in the PLA trial after baseline data collection in a randomized order. A previous study suggested that an optimal dose of caffeine (approximately 200 mg) has limited side effects on the human body and can improve attention, alertness, and cognitive processes during and following strenuous exercise [17]. The caffeine drink was prepared by grinding one caffeine tablet (ALLMAX Caffeine Tablet, HBS International Corp., ALLMAX Nutrition Inc., North York, Ontario, CA, USA) and mixing it with 100 mL of water and 0.8 g of sugar substitute sweetener (Equal Gold—Crunchy Granular Sweetener, Sanko Machinery Co., Ltd., Bangkok, Thailand) to hide the bitterness of the caffeine. The placebo drink was prepared by mixing 100 mL of water with 0.8 g of the same sugar substitute. Two drops of yellow pigment (BUTTERFLY Dark Yellow Food Coloring, Jans Enterprises Corp., El Monte, CA, USA) and a lid with a straw was used to hide potential differences in taste and color between the two drinks. The two drinks were tested by the participants in the preliminary trial, and differences were not observed between these drinks in the taste test.

### 2.3. Procedures and Exercise Protocol

Prior to the experiments, information was obtained on the participants’ personal background, taekwondo experience, daily food intake and fitness workout regimen 3 days before the experiment, and caffeine intake history. Among all the participants, 3 of them consumed coffee every day and the other 7 participants consumed coffee 3–4 times/week. The participants were required to wear sportswear and a body protector (including a full dobok, head, body, arm, shin, hand, and foot protectors) during the matches and to avoid any vigorous exercise 24 h before each main trial. A preliminary trial was conducted with the adopted experimental procedure prior to the main trials to help the participants become familiar with the procedure and cognitive function tests. The participants were prohibited from obtaining any kind of caffeine, sports supplements, or stimulants 24 h prior to the experiment regardless of their regular caffeine intake habit. Three days before the first main trial, participants were required to record their food intake. Participants were also required to repeat their own dietary records before their second main trial. To be well hydrated, all participants were instructed to drink sufficiently during the day before the main trial and ingest at least 500 mL of water on the day before the commencement of the main trials. They were also constantly reminded to drink regularly during the experiment.

As illustrated in Figure 1, in each main trial, after baseline data collection (T0) on height, body mass, heart rate (HR), Borg 6–20 rating of perceived exertion (RPE), blood lactate concentration, visual analogue scale for muscle pain (VAS-MP), and cognitive function tests—the participants were required to consume one of the two drinks in 2 min. The duration of T0 is 50 min in total. All the participants then remained seated in the lab for 60 min before completing cognitive function tests and blood lactate concentration measurements in the time period T1. The total duration of T1 is 30 min. According to a previous review [34], the plasma caffeine concentration reached a maximum level in 1 h after caffeine consumption and can be maintained for 3 to 4 h. Therefore, a waiting time of 60 min after the consumption of CAF or PLA drinks was selected to ensure sufficient time for the caffeine to be absorbed. During the waiting time, the participants watched a popular television series called “Lo and Behold”, which had neutral content [22]. The HR was recorded every 15 min during the waiting time.

Subsequently, the simulated taekwondo competition was conducted in a standard octagonal contest area after a 15-minute warm-up session that comprised of stretching and skill-based drills [35]. During the warm-up session, the participants were required to reach an HR of 160 bpm to simulate the situation of real competition, ensuring participants had sufficient warm-up before the matches begin. In the simulated taekwondo competition, two rounds of simulated matches were conducted. Each round of a simulated match lasted for 2 min, and a 1-min resting period was provided between two rounds of matches (T2). During T2, the participants were required to report their RPE and VAS-MP, and the HR was also recorded.

After the second round of the simulated match, the participants were provided a 45-min recovery period (T3). During T3, participants were required to report their RPE and VAS-MP, measure the HR and blood lactate concentrations, and complete the third set of cognitive function tests. Then all participants were required to perform a 1-min warm-up and 30-s all-out Wingate Anaerobic Test (WAnT). Verbal encouragement was provided during each WAnT. After the WAnT, the RPE, HR, VAS-MP, and blood lactate concentrations were recorded again (T4) within 1 min. The participants were also provided another 2-min cooldown. The total duration of T4 is 5 min. All the measurements in the present study covered the same training period and followed the same orders, i.e., RPE, VAS-MP, HR, and lactate were measured first, followed by the cognitive function tests. The first four measurements were completed within 1 min. The participants were allowed to leave the lab when their bodies recovered to their pre-exercise state.

### 2.4. Measurements

#### 2.4.1. Simulated Match Performance

The simulated taekwondo matches were conducted under the format used in Hong Kong competitions (two rounds of 2 min each). The competition area had an octagonal shape with a diameter of 8 m and a side length of 3.3 m. The matches were governed by qualified referees and the points were awarded according to the Olympic rules: 1 point for a valid punch to the trunk protector, 2 points for a valid kick to the trunk protector, 4 points for a valid turning kick to the trunk protector, 3 points for a valid kick to the head, 5 points for a valid turning kick to the head, and 1 point to the opponent for every penalty (known as gam-jeom). The remaining time and live match scores were shown adjacent to the competition area on iPad screens and could be observed by the participants during a match. The performance in the simulated match was evaluated in terms of the total score and winning ratio obtained by each participant.

#### 2.4.2. WAnT Performance

In the main examination session, the participants were required to perform 30 s of all-out cycling (Excalibur Sports Cycling Ergometer, Lode, Groningen, The Netherlands), and all participants executed nonstop locomotion until the end of the exercise. Before the test, the seat was adjusted according to the height of participants, and participants were required to complete 1 min of warm-up. The load of the cycling ergometer was set at 0.090 kp/kg body weight. The lactate concentration was measured within 1 min after the Wingate test using one portable lactate analyzer (Lactate Plus Meter, Nova Biomedical Corp., Waltham, MA, USA). The performance indicators included rate of fatigue, total peak and mean power, and total and mean power (wattage) per unit weight, which were recorded using a computer.

#### 2.4.3. Cognitive Function Tests

A battery of computer tests, including the Eriksen flanker test (EFT), Stroop test (ST), and rapid visual information processing test (RVIPT), were performed via a laptop computer to measure the cognitive function of the participants [36]. The order of these three tests was the same for all participants. The duration of each test was approximately 5 min, and both the accuracy, i.e., the proportion of correct responses made, and the reaction time (in ms) were used to assess the cognitive function performance. To avoid any potential learning effects, all participants were required to practice the cognitive function tests in the preliminary trial. In each main trial, participants were also asked to do several practical sessions before real tests.

In brief, the EFT assesses attention with two levels, congruent and incongruent. For this test, five arrows were shown on the computer screen in the same (congruent level) or different (incongruent) directions. Participants needed to select the arrow key pointing in the same direction as the central arrow. On both congruent and incongruent levels, the arrows were presented in green on a black background with a varied delay of 400 to 4000 ms. The arrows remained on the screen until the participant responded. The ST consisted of two levels of colors and word tests in which the target position was counterbalanced for the left and right side. The baseline level contained twenty stimuli in which the test word was placed in white in the center of the screen. The target and distractor were presented randomly on the left or right of the test word, and participants were required to respond as quickly as possible using the right or left arrow keys. The choices remained on the screen until the participants responded with an inter-stimulus interval of 1-sec. The complex level contained forty stimuli, where participants selected the color of the test words rather than the actual words. The RVIPT assesses sustained attention capacity. In this test, participants were asked to monitor consecutive sequenced numbers in a stream (100 digits per min) and tap the space bottom if three successive even (e.g., 3–5–7) or odd numbers appeared (e.g., 2–4–6). There were 8 target sequences per minute, and numbers 2–9 appeared on the screen for the duration at 600 ms intervals. Responses would be recorded within 1500 ms after the last digit sequence.

**Figure 2:**
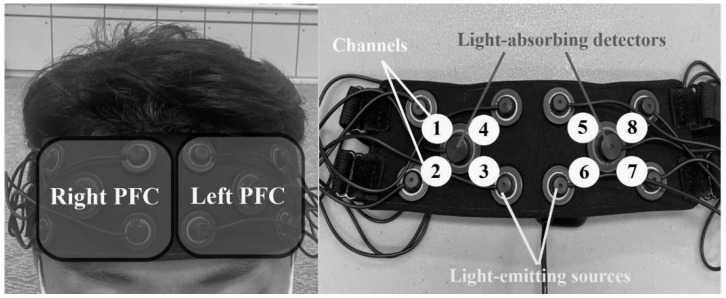
Optodes position of fNIRS device. 1–8 are channel numbers.

During the cognitive function tests, a multi-channel fNIRS system using two wavelengths of infrared light (751 and 839 nm) was adopted to scan cortical areas underlying the forehead for determining the prefrontal cortex (PFC) oxygenation levels of participants. The fNIRS (OctaMon System, Artinis Medical Systems, Gelderland, The Netherlands) device had two sets of 1 × 4 multichannel probe holders, which consisted of 8 light- emitting sources and 2 light-absorbing detectors arranged alternately at an inter-probe distance of 3.5 cm. This configuration thus resulted in 4 channels per set (a total of eight channels). Optode position was set and covered the participants’ prefrontal brain area according to the international 10–20 system with FPz located between optodes 3 and 6 (Figure 2). Through the application of light intensity on the differential path-length factor (DPF) and age-dependent DPF [37], the fNIRS device can calculate relative changes to baseline levels of H_2_Ob concentration. Before each cognitive function tests were conducted, the data of baseline condition was collected and lasted 30 to 60 s. During the fNIRS data collection process, participants were instructed to sit quietly, breathe smoothly, and avoid head movement and swallow. The HR and blood pressure were not controlled.

#### 2.4.4. HR and Blood Lactate

A Polar HR sensor (Polar Vantage NV, Polar, Kempele, Finland) was used to monitor the HR of the participants throughout the experiment. A 0.5-μL capillary blood sample was obtained through a finger prick at different time points to measure the blood lactate concentrations by using a portable blood lactate analyzer (Lactate Plus, Nova Biomedical Corp., Waltham, MA, USA).

#### 2.4.5. Subjective Measures

The RPE scale was used to measure the combination of all sensations and feelings of physical stress and general fatigue experienced by the participants during the different stages of the experiment [38]. The VAS-MP, which is a continuous scale, was used to measure muscle pain intensity [39].

### 2.5. Statistics Analysis

All data were analyzed using IBM SPSS Statistics for Windows, Version 25.0 (IBM, Armonk, NY, USA). All the results are presented in terms of the mean ± standard deviation, and *p* < 0.05 indicated statistical significance. The Shapiro Wilk tests were performed firstly for all variables to test the normality. Then the dependent measures, including cognitive function, blood lactate concentrations, HR, RPE and VAS-MP score, were analyzed using two-way repeated-measures analysis of variance (ANOVA) to determine the interactive effects and main effects of conditions (PLA vs. CAF) and time (T0, T2, T3, and T4 for blood lactate, RPE, and VAS-MP score; T0, T1 and T3 for cognitive function tests performance; T0, T1, T2, T3, and T4 for HR.) The post hoc test with Bonferroni correction was conducted if a significant interactive effect was observed. A paired sample t-test was used to compare the simulated match performance between two conditions (PLA vs. CAF). The effect sizes were estimated using either η^2^ or Cohen’s d.

The fNIRS data were collected and exported at a sample rate of 10 Hz. A moving Gaussian filter with a width of 0.3 s was applied to the data by using OXYSOFT analysis software (version 3.0.97.1, Artinis Medical Systems, Gelderland, The Netherlands) to filter out noises created physiologically (e.g., HR, respiratory and vasoconstriction), experimentally (e.g., body movement), or instrumentally. Four neighboring channels were combined to form left and right PFC areas, defined as the region of interest (ROI). The regions included the right PFC (channels 1, 2, 3, and 4) and the left PFC (channels 5, 6, 7, and 8) (Figure 2). The O_2_Hb concentration was computed and averaged at baseline and over the whole of each single cognitive test (i.e., EFT, ST, and RVIPT) at three time points (i.e., T0, T1, and T3). Similar to cognitive function tests performance, the mean O_2_Hb value of left PFC and right PFC were further analyzed using two-way repeated-measures ANOVA.

## 3. Results

### 3.1. Performance in the Simulated Matches and WAnT

Table 1 presents the scores in the simulated matches and the WAnT performance (peak power, mean power, rate of fatigue, and time to reach peak power). The total peak power (*p* = 0.007, effect size = 1.091), peak power per unit body weight (*p* = 0.018, effect size = 0.911), and mean power per unit body weight (*p* = 0.042, effect size = 0.749) were higher in the CAF trial than in the PLA trial. No differences were found between the two trials in the total scores for the simulated matches and in other WAnT performance indicators.

### 3.2. Cognitive Function Tests

The results of the cognitive function tests are presented in Table 2. No trial or time or trial × time interaction effect was found in the accuracy of all the cognitive function tests. There was a trial × time interaction effect regarding the reaction time in EFT (F = 4.137, η^2^ = 0.580, *p* = 0.013). The only trial difference was observed in the reaction time in the EFT at T1 (*p* = 0.035, effect size = 0.614). The reaction time in the EFT improved significantly in the CAF trial 1 h after caffeine ingestion (T0 vs. T1: *p* = 0.020, effect size = 0.889) and at T3 (T0 vs. T3: *p* = 0.042, effect size = 0.749). In the ST, the reaction time for the CAF trial improved significantly from T0 to T1 (*p* = 0.021, effect size = 0.887). However, at T3, the reaction time in the ST improved by a nonsignificant margin in the CAF trial (*p* = 0.051) but a significant margin in the PLA trial (*p* = 0.042, effect size = 0.509). No significant time, trial, and trial × time interaction effects were observed in the reaction time in the RVIPT.

### 3.3. fNIRS Results

**Figure 3:**
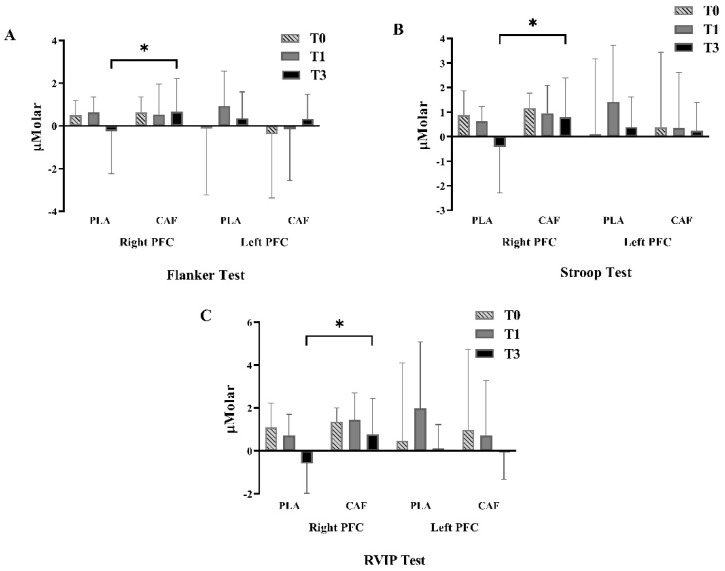
Results of fNIRS data in the left and right PFC at different time points. (**A**) Flank Test; (**B**) Stroop Test; (**C**) RVIP Test. PFC = Prefrontal cortex; PLA: Placebo trial; CAF: Caffeine trial; * Significant difference between PLA trial and CAF trial.

Only the O_2_Hb data was analyzed as alterations in O_2_Hb were considered to be reliable and sensitive to reflect cognitive performance [30]. In the EFT, there was no trial × time interaction effect in O_2_Hb concentrations in left and right PFC (*p* = 0.169 and 0.429, respectively; Figure 3A). According to pairwise comparisons, the O_2_Hb concentrations of right PFC were higher in the CAF trial than in the PLA trial at T3 (*p* = 0.048, effect size = 0.723; Figure 3A). Similarly, in the ST, there was no trial × time interaction effect in O_2_Hb concentrations in left and right PFC whereas a significant trial effect was observed in the right PFC (*p* = 0.043, effect size = 0.381; Figure 3B). Further analysis suggested that the O_2_Hb concentrations of right PFC were significantly greater at T3 in the CAF trial compared with the PLA trial (*p* = 0.021, effect size = 0.887; Figure 3B). For the RVIPT, the O_2_Hb concentrations displayed no trial × time interaction in the left and right PFC (*p* = 0.129 and 0.347, respectively; Figure 3C). Similarly, higher O_2_Hb concentrations of right PFC were observed in the CAF trial compared with that in the PLA trial at T3 (*p* = 0.011, effect size = 1.011; Figure 3C).

### 3.4. Other Indicators

**Figure 4:**
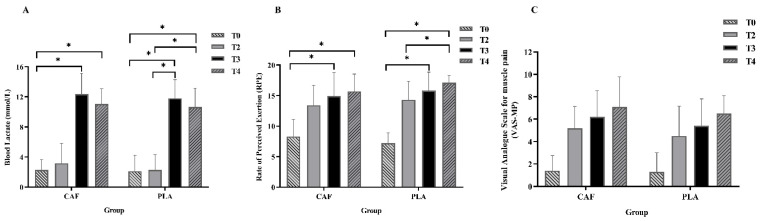
Results of blood lactate, RPE, and VAS-MP at different time points. (**A**) Blood lactate; (**B**) RPE; (**C**) VAS-MP. RPE = Rating of perceived exertion; VAS-MP = Visual Analogue Scale for muscles pain. * Significantly different between different time points (*p* < 0.05).

The results for blood lactate concentration, RPE, and VAS-MP score are displayed in Figure 4. The blood lactate concentration increased significantly at T3 and T4 after the simulated match; however, no trial differences were observed in this concentration. A significant difference was observed between the RPEs at T2 and T4 (T2 vs. T4: 14.3 ± 3.2 vs. 17.1 ± 1.2, *p* = 0.013, effect size = 0.979) in the PLA trial; however, no significant difference was observed between the RPEs at T2 and T4 in the CAF trial (T2 vs. T4: 13.4 ± 3.3 vs. 15.7 ± 2.8, *p* = 0.166). Moreover, no significant time differences were observed in the VAS-MP results for the PLA and CAF trials.

The HR results are displayed in Table 3. No trial differences were observed in the HR at each time point. The HR was higher at T2, T3, and T4 than that at T0 and T1 in both trials. Significant differences were observed in the HR between T3 and T4 (*p* = 0.000, effect size = 1.712) in the PLA trial but not in the CAF trial.

## 4. Discussion

The main findings of the present study were that compared with the placebo intake, caffeine intake increased the peak power and mean power per body weight by approximately 13% and 6%, respectively. However, the performance in the simulated match was unaffected by caffeine intake. In both trials, compared with the baseline (T0), the reaction times in the EFT reduced 1 h after caffeine consumption and after the simulated match. Immediately before exercise (T1), the reaction time in the EFT in the CAF trial was shorter than that in the PLA trial, which indicates that caffeine intake improved the EFT performance in the resting state. The fNIRS data indicated that O_2_Hb concentrations were higher in the right PFC in the CAF trial than that in the PLA trial after the simulated match (T3) in all three cognitive function tests. Moreover, although caffeine intake yielded no improvement with respect to the physiological indicators (specifically, RPE, VAS-MP score, blood lactate concentration, and HR), the RPE after the WAnT (T4) was higher than that after the first simulated match session (T2) in the PLA trial but not in CAF trial.

In the present study, caffeine intake significantly increased the anaerobic power in the WAnT (Table 1), including the peak power and mean power. The peak power and mean power in the WAnT reflect the functional capacities of the adenosine triphosphate phosphocreatine system and lactic acid system, which are the most important energy systems to determine the taekwondo performance [40]. Several studies [41,42] have suggested that caffeine ingestion can increase maximal anaerobic power, which agrees with the findings of the current study. However, not all studies support this conclusion [43,44]. A recent meta-analysis [45] of 16 studies concluded that compared with placebo ingestion, caffeine ingestion can improve peak power and mean power by 4% and 3%, respectively. Therefore, it seems that anaerobic capacity could be increased following caffeine consumption. It should be noted that, although the anaerobic capacity has improved after caffeine consumption in the present study, the performance of the simulated match, i.e., the total score and winning rate, was not different between the two trials. Therefore, the anaerobic power should not be the only factor to determine taekwondo performance. The result of the present study was consistent with one previous study [28] that also did not observe the improved performance measured by attack number or attack time after caffeine consumption in taekwondo athletes. However, another study performed among taekwondo athletes observed higher attack numbers and attack/skipping ratio, as well as the enabled maintenance of intensity after caffeine ingestion compared with placebo [24]. The inconsistency may come from different performance indicators. It should also be noted that two recent systematic reviews and meta-analyses [8,9] suggested that caffeine consumption can acutely improve performance in combat sports, although not all the included studies support this conclusion. Additionally, in the present study, the fatigue rate in WAnT did not differ between the two trials, which suggests that caffeine intake may not reduce fatigue in anaerobic exercise. Interestingly, several studies [12,24,46] have found that caffeine can reduce fatigue during endurance exercise. Thus, additional studies are required to clarify the effect of caffeine on anaerobic fatigue.

In the present study, the potential effects of caffeine intake on cognitive function were measured by three commonly used tests, i.e., EFT, ST, and RVIPT, which have been investigated to a limited extent in taekwondo athletes. The EFT is widely used to measure inhibition ability and selective attention [47]. The ST is commonly used to measure the inhibition of cognitive interference, processing speed, cognitive flexibility, and executive function [48]. The RVIPT was used to assess target sensitivity, decision-making, and sustained attention and alertness [49]. In the present study, the reaction time in the EFT and ST improved after caffeine ingestion, and this improvement was maintained after the simulated match. However, this improvement was not observed in RVIPT. In a previous study [36], following a 2.5-h exercise at 60% VO_2max_ and a time to exhaustion protocol, caffeine intake improved reaction time in both ST and RVIPT. This inconsistency can be attributed to the different exercise protocols in these two studies. An endurance exercise protocol was used in the previous study [36], whereas a protocol involving a simulated taekwondo match was adopted in the present study. The potential effect of simulated taekwondo matches on different cognitive function domains should also be considered. In previous studies [50,51], different exercise protocols and exercises with different intensities have elicited different effects on executive functions. Additionally, no significant difference was observed in the accuracy of all the cognitive tests between the CAF and PLA trials (*p* > 0.05) in the present study. One possible reason is the influence of caffeine dose. In one previous study [52], low doses of caffeine (60 mg) did not affect accuracy in cognitive function tests. More importantly, an inverted-U hypothesis has been proposed [52] regarding the effect of caffeine dose on accuracy in cognitive function tests. Thus, the dosage of caffeine used in the present study (i.e., 200 mg) may have also been insufficient for improving accuracy in the cognitive function tests.

Although the improvement in accuracy of all three cognitive function tests was not observed after caffeine consumption, the improved reaction times in the EFT and ST suggest that the adopted caffeine dose in the present study can still partially benefit cognitive function. This finding is supported by several previous studies [21,22,36], in which 200 mg of caffeine has been found to possibly benefit certain domains of cognitive function after exercise. It is well known that caffeine is a CNS stimulant that stimulates the sympathetic nerves to increase physiological arousal, increase attentional narrowing, and achieve antifatigue effects [12]. Therefore, this result indicates that caffeine intake may improve inhibition, attentional control, and cognitive flexibility to a certain degree in a simulated taekwondo match. Similarly, despite the cognitive benefits after caffeine intake, the performance of the simulated match was similar between the two trials. Therefore, cognitive function may also only partially determine taekwondo performance. Although the total score and winning rate were not improved after caffeine consumption in the present study, there is a clear trend to suggest the potential benefits of caffeine intake (PLA vs. CAF, Mean score: 16.6 ± 6.9 vs. 18.8 ± 9.0; Winning rate (%): 40.0 ± 0.5 vs. 60.0 ± 0.5). Considering the potential benefits of caffeine to anaerobic power and cognitive function, elite taekwondo athletes should be encouraged to intake some caffeine for the purpose of improving exercise performance.

The fNIRS has been increasingly applied in the fields of psychology, sports science, and neuroscience recently. In general, a simultaneous increase in O_2_Hb concentration and a decrease in HHb concentration results in increases in local arteriolar vasodilatation, cerebral blood flow, and cerebral volume, which is known as “neurovascular coupling” [53]. In the present study, the fNIRS was used to investigate the possible relationships between cognitive functions and cortical hemodynamics in the prefrontal area before and after the simulated match [30]. According to a previous study [54], the left dorsolateral prefrontal cortex and the frontopolar area may be associated with Stroop interference, and these areas can be activated by low-intensity exercise (e.g., 10-min cycling at 30% maximal oxygen consumption, VO_2max_). In the present study, however, the O_2_Hb concentrations did not increase after the simulated match (T3) in both trials. This result indicated that both left and right PFC were not affected by the simulated taekwondo match. Therefore, exercise intensity (low vs. high) and/or exercise modalities (cycling vs. taekwondo) may have different roles in determining the prefrontal hemodynamic responses. In the present study, the O_2_Hb concentrations were greater in the CAF trial than in the PLA trial in all three tests, which indicated that the right PFC was activated after caffeine ingestion and the simulated match. However, as the only difference in cognitive function performance tests between the CAF trial and PLA trial was observed in EFT before the stimulated match (T1), the cerebral hemodynamic responses should not be the only factor influencing the performance of cognitive function tests. Besides cerebral hemodynamic responses, other neurotransmitters and neurotrophic factors such as brain-derived neurotrophic factor (BDNF), insulin-like growth factor-1 (IGF-1), and vascular endothelial growth factor (VEGF) have also been suggested to promote functions and plastic changes in the brain [55]. Additionally, blood lactate concentrations have also been suggested to connect exercise and cognitive function performances [56,57], although it is not so conclusive so far. In the present study, the reaction time in EFT and ST has improved after the simulated match (T3), which is consistent with the increased blood lactate concentrations (Figure 4A). However, blood lactate concentrations did not differ between CAF and PLA trials in the present study. Furthermore, our previous study also indicated that the elevated blood lactate concentrations could not explain the differences in executive function after different types of exercise [51]. Therefore, more studies are still needed to clarify the potential mechanisms behind the benefits of caffeine consumption.

A study found that the reaction time and athletic performance in simulated taekwondo contests can be improved by caffeine intake [24]. During taekwondo matches, a low reaction time is an advantage that can help one to make better decisions regarding whether to defend or attack according to the situation and the opponent’s action. However, in the present study, caffeine intake had no effect on performance in the simulated taekwondo match. This result is consistent with that of a previous study [28], in which caffeine intake did not improve taekwondo performance with regard to the speed and frequency of attacks. Despite the differences, the mean simulated match score was not higher in the CAF trial than in the PLA trial (PLA: 16.6 ± 6.9, CAF: 18.8 ± 9.0; mean difference = 2.2 ± 9.3). Therefore, the improved reaction time and delayed fatigue caused by caffeine consumption may not benefit subsequent taekwondo performance.

No differences were observed in blood lactate concentration, HR, RPE, and VAS-MP scores between the CAF and PLA trials. However, the increase in the RPE from T2 to T4 was significant in the PLA trial but not in the CAF trial. Moreover, compared with those in PLA trials, the RPE in the CAF trial was reduced approximately by 6.2% after the first round of the simulated match (T2), 5.69% after the second round of the simulated match (T3), and 8.1% after the WAnT (T4) (Figure 4B). The result is consistent with that of a previous study [58], which found that compared with placebo ingestion, caffeine ingestion may reduce the RPE. The chemical structure of caffeine makes it act as an adenosine receptor antagonist [59], which can result in a reduction in the perception of muscle pain. However, in our study, caffeine exhibited no ability to reduce muscle pain. The recovery of HR was observed in the PLA trial (T4 vs. T2 and T3), whereas in the CAF trial, the HR exhibited a relatively steady fluctuation, without a clear presentation of autonomic HR recovery. According to one previous study [60], caffeine ingestion may delay postexercise autonomic recovery, that is, the sympathetic nervous system may be disrupted, and autonomic HR recovery may be prolonged.

In the current study, the effects of caffeine on both the simulated match performance and cognitive performance of elite taekwondo athletes were investigated, which was less reported previously. Another strength of the present study was that the fNIRS was adopted during the cognitive function tests to obtain additional physiological data for evaluating and explaining the changes in cognitive functions.

There are also several limitations that should be clearly mentioned. The first limitation of the present study was its small sample size of 10 participants due to the COVID-19 pandemic. Therefore, the statistical power might be low. Second, both males and females were included in the study. A previous study has suggested the potential differences in delayed-onset muscle soreness after caffeine metabolism between sexes [61], although this difference may not affect the anaerobic performance [62]. Therefore, further studies with large sample sizes are needed to clarify these issues. Third, the two-round match format used in this study is only adopted in Hong Kong, whereas the Olympics has three rounds. Therefore, the results of the present study may not be directly applicable to Olympic taekwondo competitions. Fourth, the lactate concentration was measured within 1 min after the WAnT which may not be the peak lactate concentration for all participants. Fifth, all participants adopted the same dosage of caffeine that did not consider the body weight of different elite taekwondo athletes. Sixth, a more carefully designed “taste” test should be performed to make sure the “double-blinded” and avoid a potential placebo effect. Additionally, participants were not required to report the potential side effects such as insomnia, nervousness, restlessness, headache, and anxiety. Considering all these limitations of the present study, it should be cautious to recommend taekwondo athletes to consume caffeine for the purpose of improving their performance in competitions.

## 5. Conclusions

In conclusion, caffeine intake enhances the anaerobic power of elite taekwondo athletes in Hong Kong. Although vague evidence supports the improved cognitive function, especially the selective attention and inhibitory function that has been observed after caffeine consumption, the fNIRS data is not consistent with the changes in cognitive function in the present study. It seems that more studies are still needed to further clarify whether and how caffeine could be consumed before taekwondo competitions to improve performance.

## Figures and Tables

**Table 1:** Results of stimulated matches score and performance of the WAnT.

	PLA	CAF	*p* Value
**Simulated matches performance**			
Mean score of stimulated matches	16.6 ± 6.9	18.8 ± 9.0	0.474
Winning rate (%)	40.0 ± 0.5	60.0 ± 0.5	0.168
**Wingate test performance**			
Peak Power (W)	1006.0 ± 366.8	1114.1 ± 325.2 **	0.007
Mean Power (W)	527.3 ± 152.7	551.2 ± 146.2	0.071
Peak Power/BW (W/kg)	14.6 ± 3.5	16.5 ± 2.7 *	0.018
Mean Power/BW (W/kg)	7.7 ± 1.2	8.2 ± 10.0 *	0.042
Rate of fatigue (%)	67.9 ± 11.5	70.6 ± 4.7	0.386
Time to Peak Power (s)	1.4 ± 0.4	1.2 ± 0.2	0.055

Note. Data are presented as means ± SD. PLA = Placebo trial; CAF = Caffeine trial; WAnT = Wingate Test. * Significantly different from PLA and CAF (*p* < 0.05). ** Significantly different from PLA and CAF (*p* < 0.01).

**Table 2:** Results of accuracy and reaction time of cognitive function test.

Cognitive Function Tests	T0		T1		T3	
	PLA	CAF	PLA	CAF	PLA	CAF
**Accuracy (%)**						
EFT	99.0 ± 1.2	98.0 ± 3.1	98.0 ± 2.1	97.7 ± 3.0	99.0 ± 1.3	98.3 ± 2.2
ST	96.5 ± 2.5	96.3 ± 4.1	98.0 ± 3.7	96.8 ± 2.1	97.0 ± 3.0	96.7 ± 3.6
RVIPT	52.7 ± 9.5	52.4 ± 18.0	56.5 ± 16.3	61.0 ± 17.4	63.4 ± 16.1	65.7 ± 22.0
**Reaction time (ms)**						
EFT	487.0 ± 63.1	494.1 ± 53.1	494.9 ± 49.2	467.9 ± 38.0 *^,a^	484.1 ± 55.6	465.4 ± 35.3 *
ST	871.3 ± 109.1	809.7 ± 101.4	811.9 ± 134.2	777.3 ± 102.8 *	778.6 ± 120.3 *	743.1 ± 146.8 ^#^
RVIPT	2021.8 ± 953.5	2404.1 ± 1811.3	1696.5 ± 867.7	1847.8 ± 1362.2	1749.6 ± 1393.1	1572.5 ± 1213.0

Note. Data are presented as means ± SD. Measurements were taken baseline (T0), before exercise (T1), and immediately after the simulated match session (T3). PLA = Placebo trial; CAF = Caffeine trial; EFT = Eriksen Flanker Test; ST = Stroop Test; RVIPT = Rapid Visual Information Processing Test. * Significant time differences from EFT-CAF trial: T0 vs. T1; T0 vs. T3. ST-CAF trial: T0 vs. T1; PLA trial: T0 vs. T3 (*p* < 0.05). # Marginal significant time differences from ST-CAF trial: T0 vs. T3 (*p* = 0.051). ^a^ Significantly different from PLA and CAF at EFT-T1 (*p* < 0.05).

**Table 3:** Results of heart rate.

	T0	T1	T2	T3	T4
PLA	80 ± 11	82 ± 7	182 ± 7 ^a,b,c^	183 ± 7 ^a,b,c^	173 ± 8 ^a,b^
CAF	86 ± 16	86 ± 15	176 ± 16 ^a,b^	178 ± 18 ^a,b^	171 ± 10 ^a,b^

Note. Data are presented as means ± SD. Measurements were taken at different time points. PLA = Placebo trial; CAF = Caffeine trial. ^a^ Significantly different from T0 (*p* < 0.05). ^b^ Significantly different from T1 (*p* < 0.05). ^c^ Significantly different from T4 (*p* < 0.05).

## Data Availability

Not applicable.

## References

[1] van Dijk G.P., Huijts M., Lodder J. (2013). Cognition Improvement in Taekwondo Novices Over 40. Results from the SEKWONDO Study. Front. Aging Neurosci..

[2] Campos F.A., Bertuzzi R., Dourado A.C., Santos V.G., Franchini E. (2012). Energy demands in taekwondo athletes during combat simulation. Eur. J. Appl. Physiol..

[3] Bridge C.A., da Silva Santos J.F., Chaabene H., Pieter W., Franchini E. (2014). Physical and physiological profiles of taekwondo athletes. Sports Med..

[4] Markovic G., Misigoj-Durakovic M., Trninic S. (2005). Fitness profile of elite Croatian female taekwondo athletes. Collegium. Antropol..

[5] Vicente-Salar N., Fuster-Munoz E., Martinez-Rodriguez A. (2022). Nutritional Ergogenic Aids in Combat Sports: A Systematic Review and Meta-Analysis. Nutrients.

[6] Smith A. (2002). Effects of caffeine on human behavior. Food Chem. Toxicol..

[7] Aguilar-Navarro M., Munoz G., Salinero J.J., Munoz-Guerra J., Fernandez-Alvarez M., Plata M.D.M., Del Coso J. (2019). Urine Caffeine Concentration in Doping Control Samples from 2004 to 2015. Nutrients.

[8] Lopez-Gonzalez L.M., Sanchez-Oliver A.J., Mata F., Jodra P., Antonio J., Dominguez R. (2018). Acute caffeine supplementation in combat sports: A systematic review. J. Int. Soc. Sports Nutr..

[9] Diaz-Lara J., Grgic J., Detanico D., Botella J., Jimenez S.L., Del Coso J. (2022). Effects of acute caffeine intake on combat sports performance: A systematic review and meta-analysis. Crit. Rev. Food Sci. Nutr..

[10] Merino-Fernández M., Giráldez-Costas V., González-García J., Gutiérrez-Hellín J., González-Millán C., Matos-Duarte M., Ruiz-Moreno C. (2022). Effects of 3 mg/kg Body Mass of Caffeine on the Performance of Jiu-Jitsu Elite Athletes. Nutrients.

[11] Fernández M.M., Ruiz-Moreno C., Giráldez-Costas V., Gonzalez-Millán C., Matos-Duarte M., Gutiérrez-Hellín J., González-García J. (2021). Caffeine Doses of 3 mg/kg Increase Unilateral and Bilateral Vertical Jump Outcomes in Elite Traditional Jiu-Jitsu Athletes. Nutrients.

[12] Doyle T.P., Lutz R.S., Pellegrino J.K., Sanders D.J., Arent S.M. (2016). The Effects of Caffeine on Arousal, Response Time, Accuracy, and Performance in Division I Collegiate Fencers. J. Strength Cond. Res..

[13] Davis J.K., Green J.M. (2009). Caffeine and anaerobic performance: Ergogenic value and mechanisms of action. Sports Med..

[14] Bazzucchi I., Felici F., Montini M., Figura F., Sacchetti M. (2011). Caffeine improves neuromuscular function during maximal dynamic exercise. Muscle Nerve.

[15] Grgic J., Grgic I., Pickering C., Schoenfeld B.J., Bishop D.J., Pedisic Z. (2020). Wake up and smell the coffee: Caffeine supplementation and exercise performance-an umbrella review of 21 published meta-analyses. Br. J. Sports Med..

[16] Magkos F., Kavouras S.A. (2005). Caffeine use in sports, pharmacokinetics in man, and cellular mechanisms of action. Crit. Rev. Food Sci. Nutr..

[17] Spriet L.L. (2014). Exercise and sport performance with low doses of caffeine. Sports Med..

[18] Brunye T.T., Mahoney C.R., Lieberman H.R., Taylor H.A. (2010). Caffeine modulates attention network function. Brain Cogn..

[19] Shabir A., Hooton A., Tallis J., Higgins M.F. (2018). The Influence of Caffeine Expectancies on Sport, Exercise, and Cognitive Performance. Nutrients.

[20] Daly J.W., Shi D., Nikodijevic O., Jacobson K.A. (1994). The role of adenosine receptors in the central action of caffeine. Pharmacopsychoecologia.

[21] Brunye T.T., Mahoney C.R., Lieberman H.R., Giles G.E., Taylor H.A. (2010). Acute caffeine consumption enhances the executive control of visual attention in habitual consumers. Brain Cogn..

[22] Giles G.E., Mahoney C.R., Brunye T.T., Taylor H.A., Kanarek R.B. (2017). Caffeine and theanine exert opposite effects on attention under emotional arousal. Can. J. Physiol. Pharmacol..

[23] McLellan T.M., Caldwell J.A., Lieberman H.R. (2016). A review of caffeine’s effects on cognitive, physical and occupational performance. Neurosci. Biobehav. Rev..

[24] Santos V.G., Santos V.R., Felippe L.J., Almeida J.W., Bertuzzi R., Kiss M.A., Lima-Silva A.E. (2014). Caffeine reduces reaction time and improves performance in simulated-contest of taekwondo. Nutrients.

[25] San Juan A.F., Lopez-Samanes A., Jodra P., Valenzuela P.L., Rueda J., Veiga-Herreros P., Perez-Lopez A., Dominguez R. (2019). Caffeine Supplementation Improves Anaerobic Performance and Neuromuscular Efficiency and Fatigue in Olympic-Level Boxers. Nutrients.

[26] Duncan M.J., Oxford S.W. (2011). The effect of caffeine ingestion on mood state and bench press performance to failure. J. Strength Cond. Res..

[27] Ali A., O’Donnell J., Von Hurst P., Foskett A., Holland S., Starck C., Rutherfurd-Markwick K. (2016). Caffeine ingestion enhances perceptual responses during intermittent exercise in female team-game players. J. Sports Sci..

[28] Lopes-Silva J.P., Silva Santos J.F., Branco B.H., Abad C.C., Oliveira L.F., Loturco I., Franchini E. (2016). Correction: Caffeine Ingestion Increases Estimated Glycolytic Metabolism during Taekwondo Combat Simulation but Does Not Improve Performance or Parasympathetic Reactivation. PLoS ONE.

[29] Martins G.L., Guilherme J., Ferreira L.H.B., de Souza-Junior T.P., Lancha A.H. (2020). Caffeine and Exercise Performance: Possible Directions for Definitive Findings. Front. Sports Act. Living.

[30] Herold F., Wiegel P., Scholkmann F., Muller N.G. (2018). Applications of Functional Near-Infrared Spectroscopy (fNIRS) Neuroimaging in Exercise(-)Cognition Science: A Systematic, Methodology-Focused Review. J. Clin. Med..

[31] Albinet C.T., Mandrick K., Bernard P.L., Perrey S., Blain H. (2014). Improved cerebral oxygenation response and executive performance as a function of cardiorespiratory fitness in older women: A fNIRS study. Front. Aging Neurosci..

[32] Gary R.A., Brunn K. (2014). Aerobic exercise as an adjunct therapy for improving cognitive function in heart failure. Cardiol. Res. Pract..

[33] Yuan Y., Li G., Ren H., Chen W. (2020). Caffeine Effect on Cognitive Function during a Stroop Task: fNIRS Study. Neural Plast..

[34] Graham T.E. (2001). Caffeine and exercise: Metabolism, endurance and performance. Sports Med..

[35] Alp M., Çatıkkaş F., Kurt C. (2018). Acute effects of static and dynamic stretching exercises on lower extremity isokinetic strength in taekwondo athletes. Isokinet. Exerc. Sci..

[36] Hogervorst E., Bandelow S., Schmitt J., Jentjens R., Oliveira M., Allgrove J., Carter T., Gleeson M. (2008). Caffeine improves physical and cognitive performance during exhaustive exercise. Med. Sci. Sports Exerc..

[37] Duncan A., Meek J.H., Clemence M., Elwell C.E., Fallon P., Tyszczuk L., Cope M., Delpy D.T. (1996). Measurement of cranial optical path length as a function of age using phase resolved near infrared spectroscopy. Pediatr. Res..

[38] Williams N. (2017). The Borg Rating of Perceived Exertion (RPE) scale. Occup. Med..

[39] Goddard G., Karibe H., McNeill C. (2004). Reproducibility of visual analog scale (VAS) pain scores to mechanical pressure. Cranio.

[40] Chtourou H., Zarrouk N., Chaouachi A., Dogui M., Behm D.G., Chamari K., Hug F., Souissi N. (2011). Diurnal variation in Wingate-test performance and associated electromyographic parameters. Chronobiol. Int..

[41] Bell D.G., Jacobs I., Ellerington K. (2001). Effect of caffeine and ephedrine ingestion on anaerobic exercise performance. Med. Sci. Sports Exerc..

[42] Glaister M., Muniz-Pumares D., Patterson S.D., Foley P., McInnes G. (2015). Caffeine supplementation and peak anaerobic power output. Eur. J. Sport Sci..

[43] Hoffman J.R., Kang J., Ratamess N.A., Jennings P.F., Mangine G.T., Faigenbaum A.D. (2007). Effect of nutritionally enriched coffee consumption on aerobic and anaerobic exercise performance. J. Strength Cond. Res..

[44] Souissi Y., Souissi M., Chtourou H. (2019). Effects of caffeine ingestion on the diurnal variation of cognitive and repeated high-intensity performances. Pharmacol. Biochem. Behav..

[45] Grgic J. (2018). Caffeine ingestion enhances Wingate performance: A meta-analysis. Eur. J. Sport Sci..

[46] Doherty M., Smith P.M. (2004). Effects of caffeine ingestion on exercise testing: A meta-analysis. Int. J. Sport Nutr. Exerc. Metab..

[47] Chan A.S., Lee T.L., Yeung M.K., Hamblin M.R. (2019). Photobiomodulation improves the frontal cognitive function of older adults. Int. J. Geriatr. Psychiatry.

[48] Erdodi L.A., Sagar S., Seke K., Zuccato B.G., Schwartz E.S., Roth R.M. (2018). The Stroop Test as a Measure of Performance Validity in Adults Clinically Referred for Neuropsychological Assessment. Psychol. Assess..

[49] Corr P.J. (2002). JA Gray’s reinforcement sensitivity theory: Tests of the joint subsystems hypothesis of anxiety and impulsivity. Personal. Individ. Differ..

[50] Tsukamoto H., Suga T., Takenaka S., Tanaka D., Takeuchi T., Hamaoka T., Isaka T., Hashimoto T. (2016). Greater impact of acute high-intensity interval exercise on post-exercise executive function compared to moderate-intensity continuous exercise. Physiol. Behav..

[51] Zhu Y., Sun F., Chiu M.M., Siu A.Y. (2021). Effects of high-intensity interval exercise and moderate-intensity continuous exercise on executive function of healthy young males. Physiol. Behav..

[52] Durlach P.J. (1998). The effects of a low dose of caffeine on cognitive performance. Psychopharmacology.

[53] Ferrari M., Quaresima V. (2012). A brief review on the history of human functional near-infrared spectroscopy (fNIRS) development and fields of application. Neuroimage.

[54] Byun K., Hyodo K., Suwabe K., Ochi G., Sakairi Y., Kato M., Dan I., Soya H. (2014). Positive effect of acute mild exercise on executive function via arousal-related prefrontal activations: An fNIRS study. Neuroimage.

[55] Cotman C.W., Berchtold N.C., Christie L.A. (2007). Exercise builds brain health: Key roles of growth factor cascades and inflammation. Trends Neurosci..

[56] Coco M., Buscemi A., Cavallari P., Massimino S., Rinella S., Tortorici M.M., Maci T., Perciavalle V., Tusak M., Di Corrado D. (2020). Executive Functions during Submaximal Exercises in Male Athletes: Role of Blood Lactate. Front. Psychol..

[57] Kujach S., Olek R.A., Byun K., Suwabe K., Sitek E.J., Ziemann E., Laskowski R., Soya H. (2019). Acute Sprint Interval Exercise Increases Both Cognitive Functions and Peripheral Neurotrophic Factors in Humans: The Possible Involvement of Lactate. Front. Neurosci..

[58] Pomportes L., Brisswalter J., Hays A., Davranche K. (2019). Effects of Carbohydrate, Caffeine, and Guarana on Cognitive Performance, Perceived Exertion, and Shooting Performance in High-Level Athletes. Int. J. Sports Physiol. Perform..

[59] Ribeiro J.A., Sebastiao A.M. (2010). Caffeine and adenosine. J. Alzheimers Dis..

[60] Bunsawat K., White D.W., Kappus R.M., Baynard T. (2015). Caffeine delays autonomic recovery following acute exercise. Eur. J. Prev. Cardiol..

[61] Chen H.Y., Chen Y.C., Tung K., Chao H.H., Wang H.S. (2019). Effects of caffeine and sex on muscle performance and delayed-onset muscle soreness after exercise-induced muscle damage: A double-blind randomized trial. J. Appl. Physiol..

[62] Chen H.Y., Wang H.S., Tung K., Chao H.H. (2015). Effects of Gender Difference and Caffeine Supplementation on Anaerobic Muscle Performance. Int. J. Sports Med..

